# Development and Application of a Structural Health Monitoring System Based on Wireless Smart Aggregates

**DOI:** 10.3390/s17071641

**Published:** 2017-07-17

**Authors:** Shi Yan, Haoyan Ma, Peng Li, Gangbing Song, Jianxin Wu

**Affiliations:** 1 School of Civil Engineering, Shenyang Jianzhu University, Shenyang 110168, China; mahaoyan2012@163.com; 2 Department of Mechanical Engineering, University of Houston, Houston, TX 77004, USA; pli7@uh.edu (P.L.); gsong@uh.edu (G.S.); 3 Liaoning Electric Power Survey & Design Institute, Shenyang 110179, China; wujianxin@lepdi.com.cn

**Keywords:** structural health monitoring (SHM), concrete crack detection (CCD), piezoelectric ceramics, wireless smart aggregates, Zigbee, active wave based method

## Abstract

Structural health monitoring (SHM) systems can improve the safety and reliability of structures, reduce maintenance costs, and extend service life. Research on concrete SHMs using piezoelectric-based smart aggregates have reached great achievements. However, the newly developed techniques have not been widely applied in practical engineering, largely due to the wiring problems associated with large-scale structural health monitoring. The cumbersome wiring requires much material and labor work, and more importantly, the associated maintenance work is also very heavy. Targeting a practical large scale concrete crack detection (CCD) application, a smart aggregates-based wireless sensor network system is proposed for the CCD application. The developed CCD system uses Zigbee 802.15.4 protocols, and is able to perform dynamic stress monitoring, structural impact capturing, and internal crack detection. The system has been experimentally validated, and the experimental results demonstrated the effectiveness of the proposed system. This work provides important support for practical CCD applications using wireless smart aggregates.

## 1. Introduction

Concrete structures, such as civil infrastructures, residential buildings, and industrial structures, are possibly the most common structures. However, the health of concrete structures is constantly affected and degraded throughout their service life by various kinds of factors, such as aging, fatigue, corrosion, and even natural disasters. The accumulated degradation will reduce the structures’ disaster resistance ability, and can sometimes lead to partial failure, failure, or even the complete collapse of the structures. The potential for such accidents is a direct threat to the safety of both lives and properties. With these rising concerns, health monitoring technology of concrete structures has become one of the top research focus areas in recent years. Among them, the use of piezoceramic transducers for the health monitoring of concrete structures has achieved good initial results.

At present, piezoelectric-based structural health monitoring technology can be classified into two main categories: active monitoring and passive monitoring. Based on the damage diagnosis methods, active monitoring technology can also be divided into the stress wave-based method and the mechanical impedance-based method. The basic principle of the wave-based method is to use the active sensing property of piezoelectric ceramic to establish the actuator-sensor arrays inside structures. Through scanning and analyzing the difference between the original signal and the current one, the identification and diagnosis of structural damages can be achieved [1,2,3,4,5,6,7,8,9,10,11]. In recent years, a new type of sensor, smart aggregate, that uses an embedded piezoceramic patch in a small concrete block similar to a real aggregate has been used to monitor the health of concrete structures [12,13,14,15,16,17]. With the smart aggregates, the development of concrete structure cracks can be effectively detected and monitored using an active sensing-based monitoring method. The mechanical impedance method is commonly used for the monitoring of a structure’s local damage, mainly through comparing the mechanical impedance value of the damaged structure with the healthy one [18,19,20,21,22].

The passive monitoring technology utilizes the high-impedance characteristics of piezoceramics to monitor the dynamic responses of the structures. The basic principle of the impedance-based structural health monitoring method is to excite the lead zirconate titanate (PZT) sensors attached to the host structure, and to measure the corresponding electrical impedance variation induced by any structural damage [23]. At present, applications using the passive monitoring method include structural vibration condition monitoring, impact load monitoring, and structural monitoring based on an acoustic emission technique. In 2006, Yang used this method to monitor the deformation of a two story reinforced concrete frame structure under earthquake ground motion [24]. The test results showed that the piezoelectric ceramic can effectively monitor stress changes in the structure. In 2007, Song et al. developed a piezoceramic-based method to monitor over-height trucks hitting a bridge [25].

Traditional structural health monitoring (SHM) technology usually uses a wired network for data collection. The advantage of such systems is its good anti-interference ability. However, such wired systems require the heavy use of cables and much manpower, with an increased wiring complexity and maintenance cost as the number of nodes increases. Also, in certain situations, it may not be possible to install cables for such monitoring. With the advancement of wireless communication technology, wireless sensor networks (WSNs) have become mature and have been applied to SHM [26,27,28,29]. Compared with the wired SHM systems, the WSN-based systems provided a much lower system and maintenance cost, and they greatly boosted the practical use of SHM for large scale structures.

This paper develops a wireless smart aggregates (WSAs) based concrete crack detection (CCD) system targeting practical large scale applications. The developed CCD system is capable of monitoring the stress and identifying cracks inside the concrete structures. The developed system eliminates the need for wiring from sensors to the central controller, which makes it suitable for complex monitoring conditions, such as the SHM of large scale concrete structures. The system has been experimentally validated, and the experimental results demonstrated the effectiveness of the proposed system. The research findings help to promote the development of smart aggregate-based CCD technology from the experimental research stage to the engineering application stage.

## 2. Design of the Wireless Smart Aggregate SHM System

### 2.1. System Setup and Signal Flow

**Figure 1:**
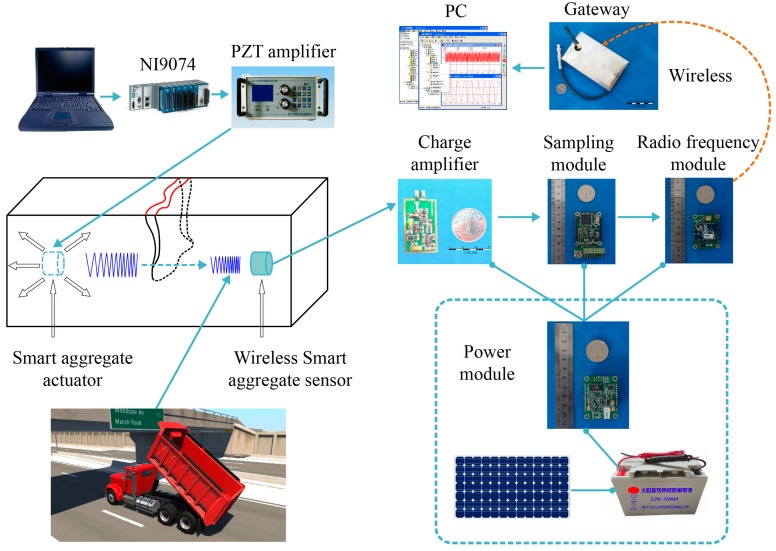
Overview of system setup and signal flow of the structural health monitoring (SHM) system. PZT, lead zirconate titanate; PC, personal computer.

Figure 1 provides an overview of the wireless smart aggregate SHM system setup and its signal flow. The developed system is comprised of a detected reinforced concrete (RC) structure, a signal excitation module, a signal data acquisition module, a wireless communication module, and a power module. The smart aggregates are pre-embedded in the detected RC structure. For the signal excitation module, the NI9074 [30] and a power amplifier are used to generate the excitation signal on the actuating smart aggregate. For the signal data acquisition module, monitoring signals from stress waves received by the sensor smart aggregates are converted into charge signals. Then, the charge amplifier, whose conversion rate is 0.1 mV/pc, will convert the charge signals to voltage signals and the amplified signals are transmitted to the sampling module. For the wireless communication module, with the analog/digital (A/D) converter, the sampling module converts the received voltage signals into digital binaries, and transmits them to the gateway through the Zigbee wireless network using the radio frequency (RF) module. The gateway receives binary digital signals from each WSA monitoring node, and transmits them to a personal computer (PC) for signal processing. The whole system is powered by the power module.

### 2.2. Selection of Wireless Network

Currently, many types of wireless technologies are available. Table 1 provides a comparison among the most commonly used wireless technologies, namely Zigbee, Bluetooth, Wi-Fi, and mobile communications. Although Wi-Fi has a high data transfer rate, it is not suitable for long-term SHM due to its high power consumption. Bluetooth also has a relatively high data transfer rate. However, the effective transmission distance is short (about 10 m). Mobile communication technology covers long distance communications. However, the service fees increase the cost for practical large scale SHM. Zigbee, based on the IEEE802.15.4 protocol, has many advantages over other wireless technologies for SHM-type applications, such as self-organization, low power, low cost, and high network capacity. These advantages make Zigbee a preferred wireless network for SHM. Moreover, smart aggregate-based SHM requires a relatively high data rate to cover the high bandwidth features of PZTs. This is a challenge given the limited computational powers of the microcontrollers and the limited power budget from the batteries. This paper develops a Zigbee-based wireless smart aggregate CCD system that solves such conflict between the power and data rates, and hence enables to be a high sensitive device for large scale concrete SHM.

### 2.3. WSA Monitoring Nodes

The WSA health monitoring node is built with two main parts: the piezoceramic smart aggregates and the signal transceiver module. The piezoceramic smart aggregates are used to perform active sensing. They can be used to generate excitations and send out stress waves across the concrete structure, and can also be used to sense the propagated stress waves for concrete health analysis. The piezoelectric smart aggregate has the distinct properties of sensing and actuating dual-functions, a wide frequency band, quick responses, a good combination effect with concrete, a low cost, and a long service time [12,13,14,15,16,17]. These properties of the smart aggregate are obviously different from strain sensing cement-based materials doped with carbon nanotubes in fabrication. The smart aggregates need a waterproof and insulating glue layer in fabrication to prevent the embedded PZT chip from being damaged for it to play a role in producing piezoelectric effects for damage identification or SHM. The strain sensing cement-based materials’ key technique in fabrication is how to disperse carbon nanotubes in the cement-based materials to ensure the electrical conductivity of the composite for SHM [31,32].

**Figure 2:**
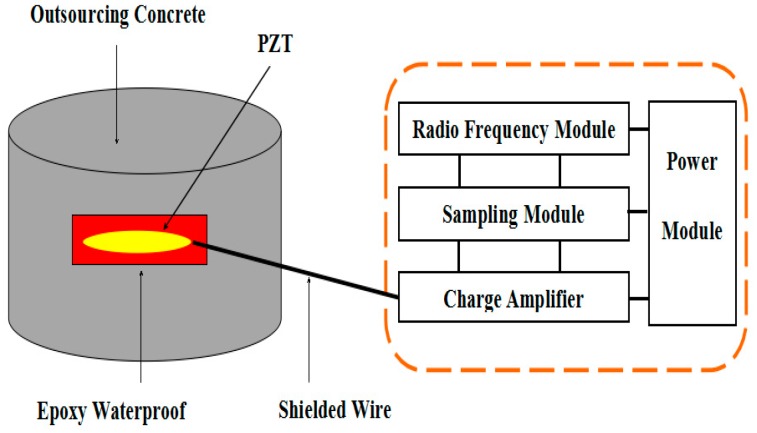
Health monitoring node based on wireless smart aggregates (WSAs).

**Figure 3:**
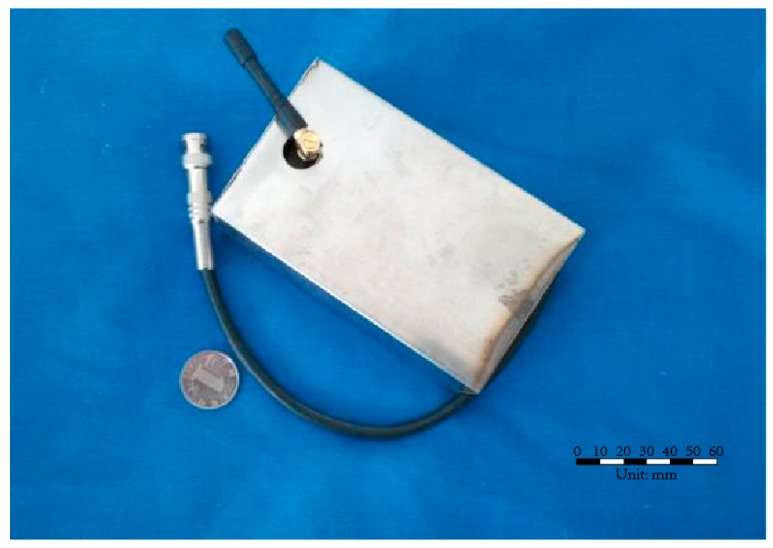
A Health monitoring node used in the experiment.

The signal transceiver module is used to acquire the stress wave signals from the smart aggregate and transmit the result wirelessly to the station. The structure of the WSA node is illustrated in Figure 2. The monitoring nodes are encapsulated in white steel protection shells, with dimensions of 150 mm × 100 mm × 50 mm, as shown in Figure 3.

**Figure 4:**
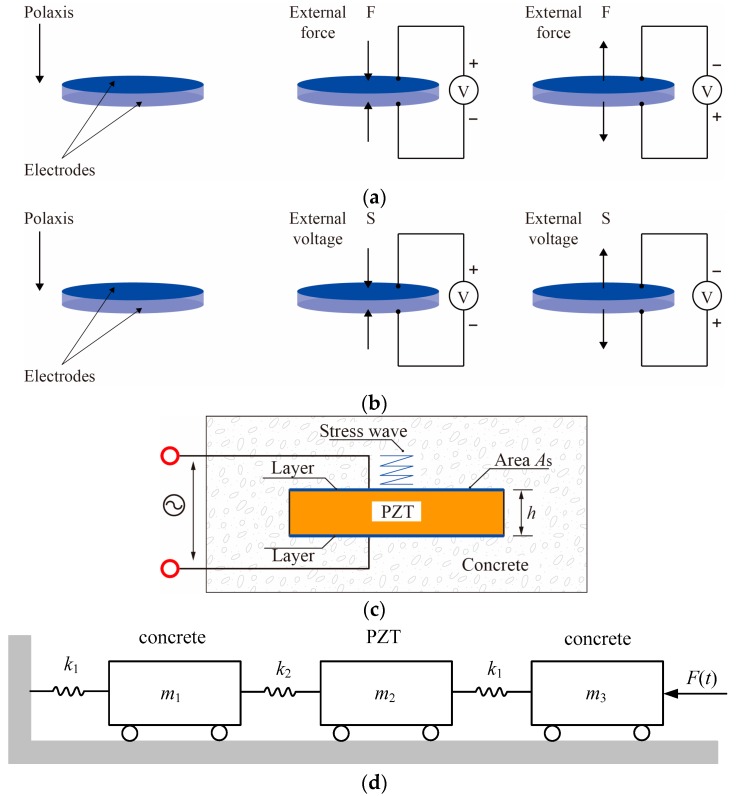
Piezoelectric effects and smart aggregate function. (**a**) Direct piezoelectric effect (sensing); (**b**) Inverse piezoelectric effect (actuating); (**c**) Smart aggregate model; (**d**) Mechanics model of the embedded PZT sensor.

The smart aggregate is an aggregate with an embedded piezoceramic disk [33,34]. The function of a piezoceramic is illustrated in Figure 4. When the piezoceramic disk inside the smart aggregate is compressed or stretched, the mechanical energy will be transformed into electrical charges, and this piezoelectric effect can be used as a sensor to sense stresses, shown in Figure 4a. On the other hand, under a changing electric field, the piezoelectric ceramic can convert the electric energy into mechanical vibration, where the smart aggregate can be used as actuator to produce excitations, shown in Figure 4b. The mechanical function of the smart aggregate, shown in Figure 4c, can be described as a PZT chip protected by two thin layers and embedded in concrete. As a stress wave is applied from the concrete to the layer, the PZT chip can be compressed or stretched, and an electrical signal will be generated; on the contrary, as an electrical field is applied on the PZT chip, it will generate a stress wave which will propagate in the host concrete, shown in Figure 4d. The orientation of the smart aggregate is defined in the same way as the PZT disk. Therefore, the smart aggregate is designed and installed at the given position, which makes the orientation of the smart aggregate perpendicular to the assumed concrete crack direction.

**Figure 5:**
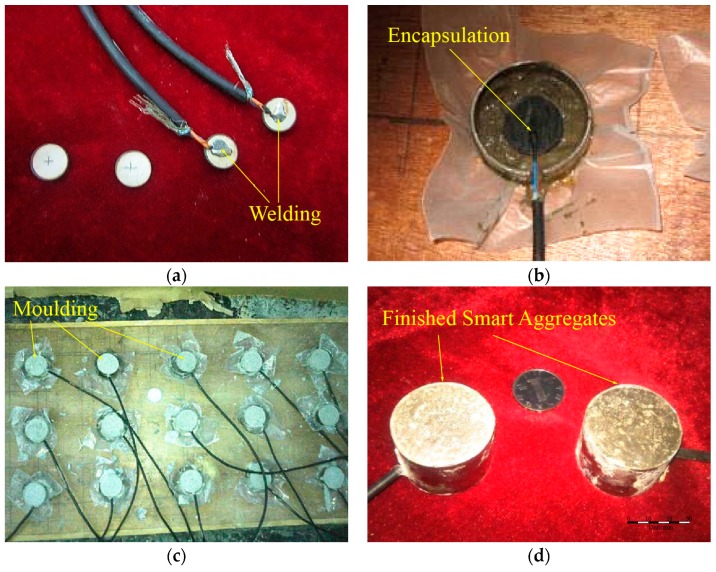
Production process of smart aggregates. (**a**) Welding; (**b**) Encapsulation; (**c**) Moulding; (**d**) Finished smart aggregates.

The PZT type of piezoceramic is used in this research to build the smart aggregates due to its strong piezoelectric effect. In this paper, PZT–4, with high sensitivity, and applying the transverse piezoelectric effect and orientation of d_33_, is used. The fabrication process of the smart aggregate is illustrated step by step in Figure 5. The main steps include the soldering of electric wires onto the PZT disk, waterproofing the PZT disk, installing the PZT disk into a mold, pouring the concrete, and removing the mold.

**Figure 6:**
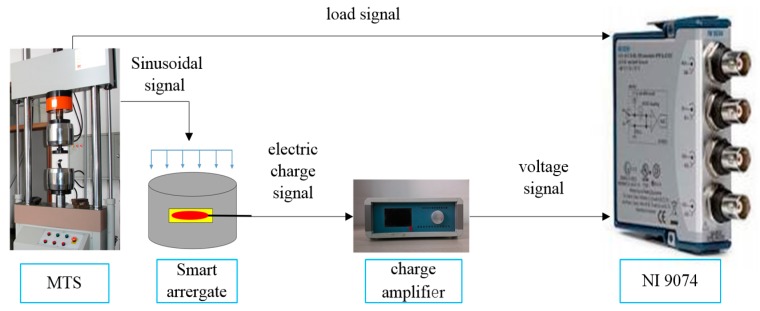
Calibration of smart aggregates.

**Figure 7:**
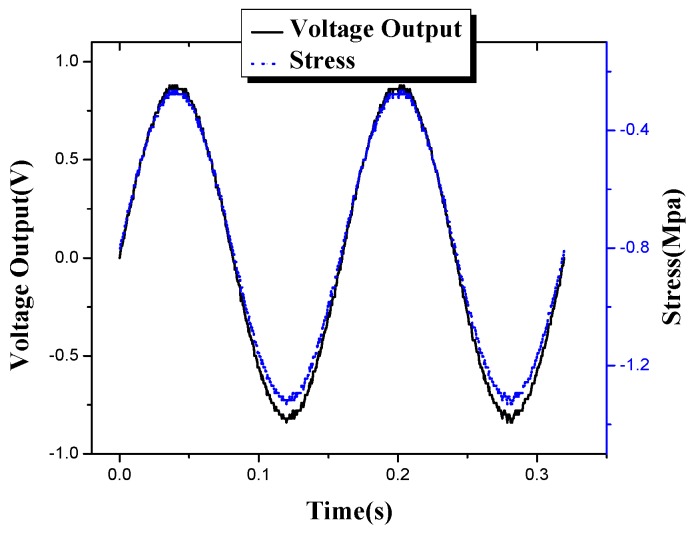
Stress of smart aggregate vs. voltage output at a circle of 0.16 s.

**Figure 8:**
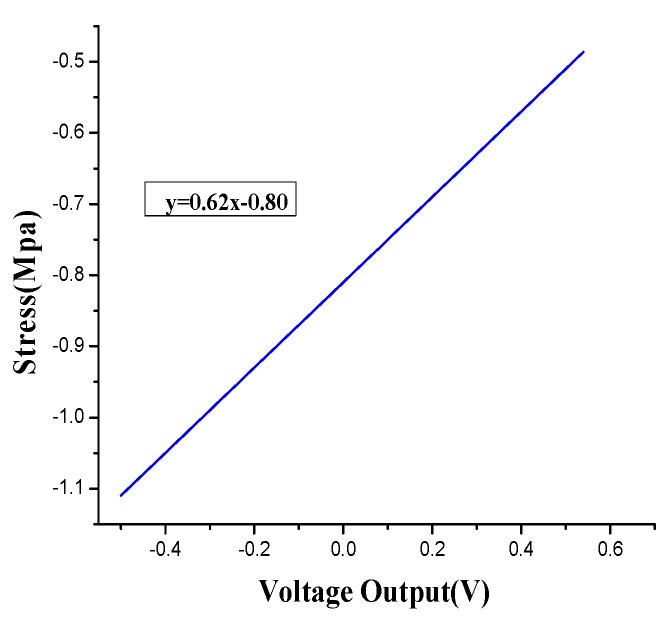
The fitting line at a circle of 0.16 s.

A calibration test is performed after the concrete is cured to obtain the sensitivity coefficient of each smart aggregate. The electro-hydraulic servo testing machine MTS (CSS-280I-250) is used to calibrate PZT-based smart aggregates. The charge amplifier from Hengke (HK9038) is selected to perform signal conditioning. The signal is recorded using the national instrument data acquisition system (NI9234) [35]. Figure 6 illustrates the calibration process. By applying sinusoidal loads at different frequencies on the smart aggregates, the output from the smart aggregate is correlated with the load applied. Figure 7 shows a case where a sinusoidal load of 10 Hz is applied on the wireless smart aggregates, and the correlation of the wireless smart aggregate’s voltage output and the applied MTS load is shown in Figure 8. It can be seen that the smart aggregate bears an almost linear correlation with the load applied. The slope of the curve in Figure 8 represents the sensitivity coefficient of the wireless smart aggregate.

The signal transceiver module mainly includes four sub-modules: a wireless radio module, a power supply module, a data acquisition module, and a charge amplifier module. Each sub-module is a separate printed circuit board (PCB).

**Figure 9:**
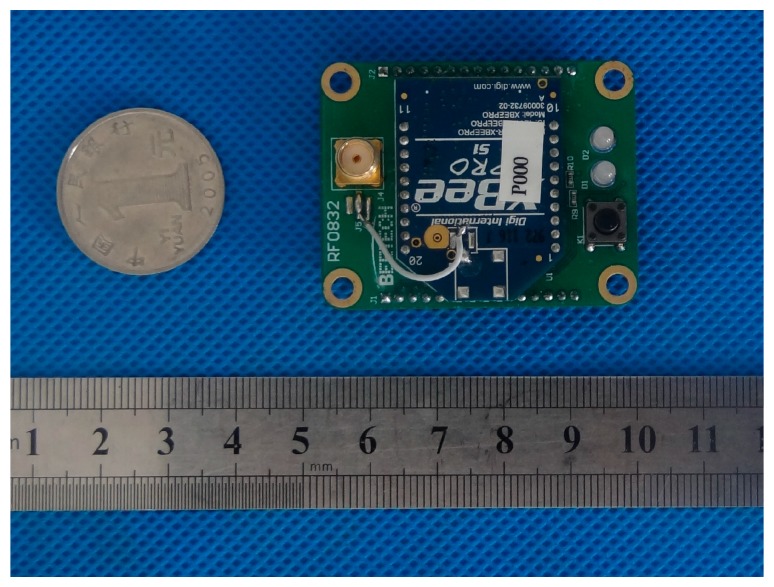
Wireless radio frequency module.

For the wireless radio module, the RF chip MC13193 from Freescale is chosen as the solution for the single chip Zigbee implementation, as shown in Figure 9. Code warrior is used as the software development platform for this microprocessor.

**Figure 10:**
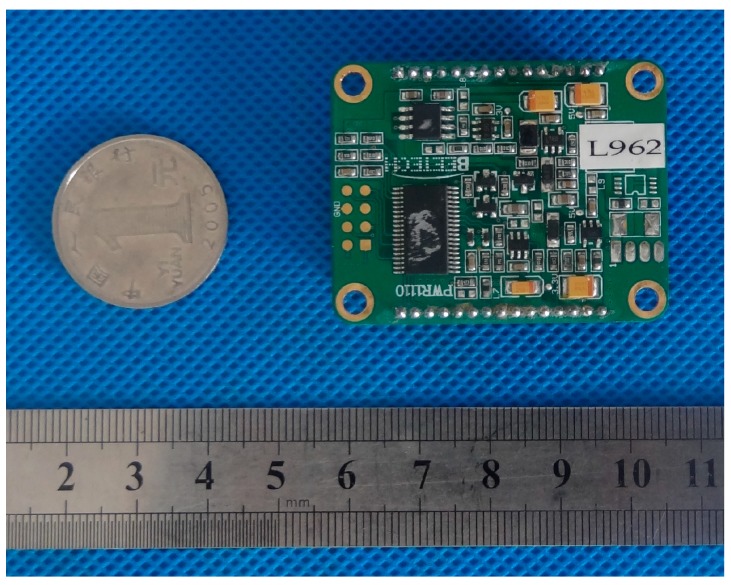
Power module.

For the power module, the MSP430F2 chip was used as a microcontroller to perform battery monitoring and control. A lithium battery with a capacity of 1000 mAh was chosen. The PCB is shown in Figure 10. The MSP430 series was chosen due to its advantages in power saving. The corresponding software platform is IAR EW for MSP430.

**Figure 11:**
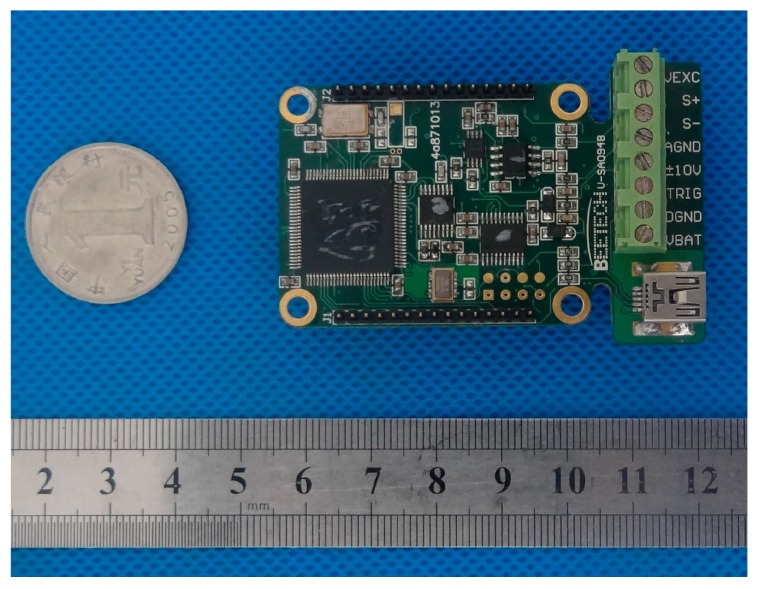
Sampling module.

As for the sampling module, the STM32F103 series chips were chosen, as shown in Figure 11. The STM32F103 is cost-effective and rapid-responding. The corresponding software platform is EWARM.

**Figure 12:**
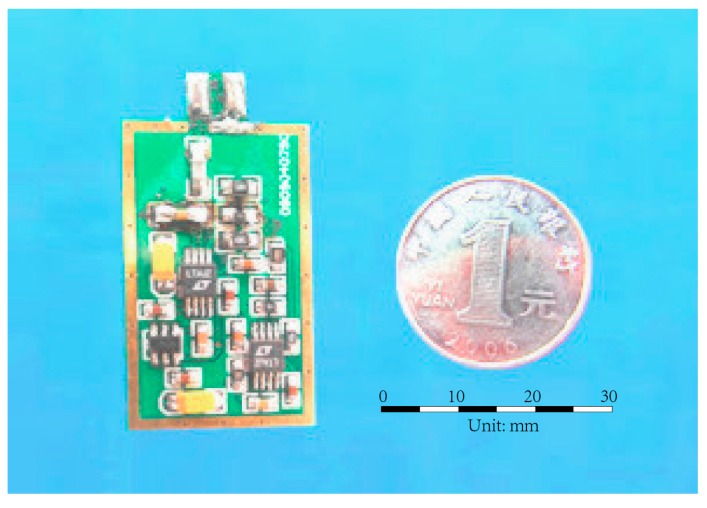
Charge amplifier.

**Figure 13:**
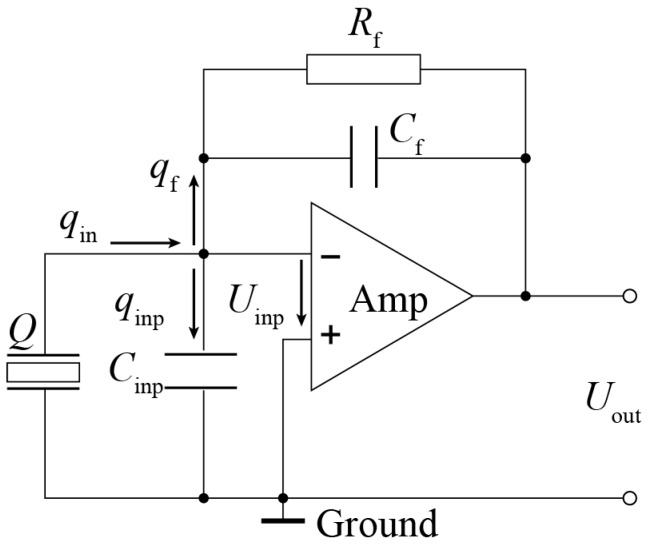
The charge amplifier.

As for the charge amplifier, the single-channel charge amplifier produced by Beijing Bichuang Company of China was used, as shown in Figure 12. The conversion coefficient is 0.1 mv/pc. The simplified circuit diagram is shown in Figure 13, where *Q* is the charge quantity produced by the smart aggregate from external force; *q*_in_ is the quantity of electric charge of input node; *q*_inp_ is the quantity of electric charge at the charge amplifier input capacitance; *q*_f_ is the quantity of electric charge at the feedback capacitor; *C*_inp_, *C*_f_, *R*_f_, *U*_inp_, and *U*_out_ are the input capacitance, the feedback capacitance, the feedback resistance, the input voltage, and the output voltage of the charge amplifier, respectively. According to the node charge conservation theorem, the input and output charge at every node must be the same, as shown in Equations (1) and (2). In normal operation conditions, the input potential difference of in-phase and phase inversion of operational amplifier is zero; therefore, the input voltage of charge amplifier *U*_inp_ is the grounding voltage, namely *U*_inp_ = 0. Equation (2) can be simplified to Equation (3); then, the output voltage of the operational amplifier can be obtained. As shown in Equation (4), for a given charge amplifier, the feedback capacitance is constant, and the output voltage of the charge amplifier is proportional to the quantity of electric charge produced by the piezoelectric sensing element without attenuation and lag phenomenon. Since the quantity of electric charge produced by the piezoelectric sensor is proportional to the external force, the output voltage of the charge amplifier will also be proportional to the external force, which defines the basic principle for smart aggregate-based health monitoring.

(1) $$q_{\mathrm{in}}=q_{lip}+q_{f}$$

(2) $$q_{\mathrm{in}}=U_{\mathrm{inp}}C_{inp}+U_{f}C_{f}$$

(3) $$q_{\mathrm{in}}=U_{f}C_{f}$$

(4) $$U_{\mathrm{out}}=U_{f}=\frac{q_{in}}{C_{f}}=\frac{Q}{C_{f}}$$

The sampling frequency of the wireless sensor used in the experiment is 1 kHz, and the capacity of the wireless sensor is 2 GB. If it is necessary, the data can be uploaded to the gateway directly.

### 2.4. Auxiliary Equipment

To meet the needs of different monitoring functions, the developed system is also equipped with some auxiliary equipment. A function generator, a power amplifier, a solar-cell panel and a rechargeable battery, whose capacity is 50 Ah and nominal voltage is 12 V, are also used for excitation signal generation, amplification, extra power supply, and power storage, respectively.

The power amplifier and function generator are only used in the active monitoring mode. The power amplifier is used to amplify the excitation signals generated from function generator. Considering the fact that line power may not be available in the monitoring field, the system is also equipped with a solar-cell panel and an accumulator.

## 3. Active Monitoring Mode

Two kinds of passive and active monitoring modes are used to validate the effectiveness of the proposed WSAs-based SHM system. The proposed passive mode is validated by an internal load monitoring test of a concrete bridge model under impact loading [36]. The experimental results show that the passive mode in the developed wireless system is stable and reliable, and can be applied in concrete bridge’s structure health monitoring under impact loading. This paper focuses on the validation of the active monitoring mode.

### 3.1. Active Monitoring Method

The active monitoring mode is based on wave-based active monitoring technology to detect cracks inside a concrete structure. The basic principle is that the smart aggregates, which are used as transducers, are embedded in different locations inside the concrete structure. The actuator generates an excitation signal, which propagates through the concrete structure reaching the sensor receiving side. Based on the analysis of the changes in the signal and the extraction of the damage factor, the on-line system for the diagnosis of the crack damage in concrete structures can be realized. The hardware of the module includes a function generator, a power supply, WSA nodes, and a computer, respectively.

According to the results of the research by [25], the amplitude of the analyzed signal is sensitive to the crack damage in the concrete structure. The attenuation in the amplitude will increase as the damage becomes more severe. Therefore, the signal amplitude can be used as a damage identification parameter. It can also be referred to as the energy value through quantification. The energy value represented by the absolute value of the square integral is the amplitude of the signal after discretization. A damage index can be defined by Equations (5)–(7). In the equations, *E*_j_ is the energy value at a moment, as shown in Equation (5); and *x*_i_ is the discrete signal at each collecting point by the sensors. If *E*_h_ is the energy value in a healthly state, then the relative state of the structure can be expressed by Equation (6). *x*_h_(*n*) and *x*_i_(*n*) are discrete signals in the healthly and damaged states, respectively, which are collected by the sensor.

(5) $$E_{i}=\sum_{n=1}^{m}{\left|x_{i}(n)\right|}^{2}=x_{i}{\left(1\right)}^{2}+\cdots +x_{i}{\left(n\right)}^{2}+\cdots +x_{i}{\left(m\right)}^{2}$$

(6) $$H_{i}=\frac{E_{i}}{E_{h}}=\frac{\sum_{n=0}^{\infty }{\left|x_{i}(n)\right|}^{2}}{\sum_{n=0}^{\infty }{\left|x_{h}(n)\right|}^{2}}\times 100\%$$

(7) $${D=1-H}_{i}=1-\frac{E_{i}}{E_{h}}=1-\frac{\sum_{n=0}^{\infty }{\left|x_{i}(n)\right|}^{2}}{\sum_{n=0}^{\infty }{\left|x_{h}(n)\right|}^{2}}\times 100\%$$

As shown in Equation (7), it is easy to observe that, when the monitored member is in a healthy condition, *H*_*i*_ = 1; when the monitored member is severely damaged, *H*_*i*_ tends to zero. In the actual health monitoring, the more severe the damage is, the higher the damage index the structure will have. Therefore, selecting *D* values as damage indexes in this paper is meaningful.

### 3.2. Validation Test of Active Monitoring Function

**Figure 14:**
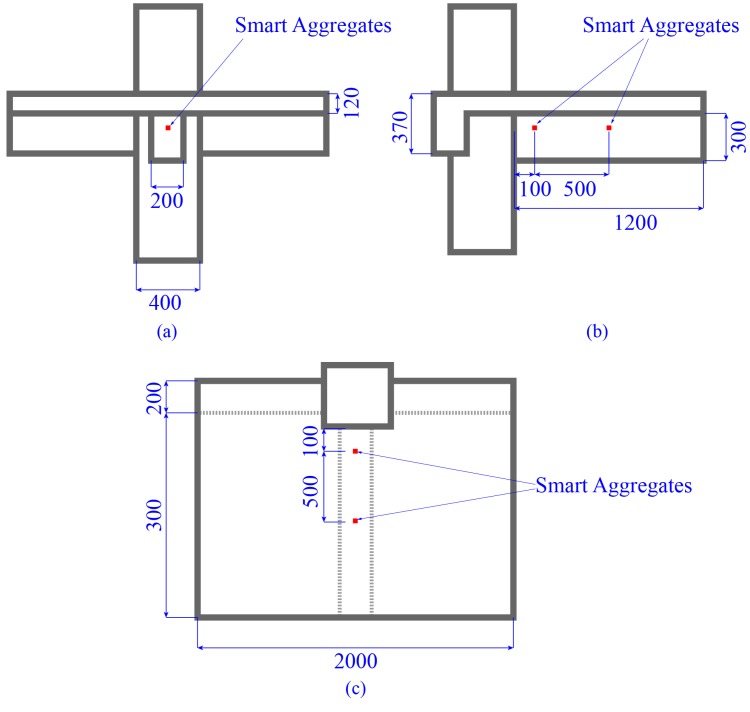
The size of the test specimen (unit: mm). (**a**) Front view; (**b**) Side view; (**c**) Top view.

**Figure 15:**
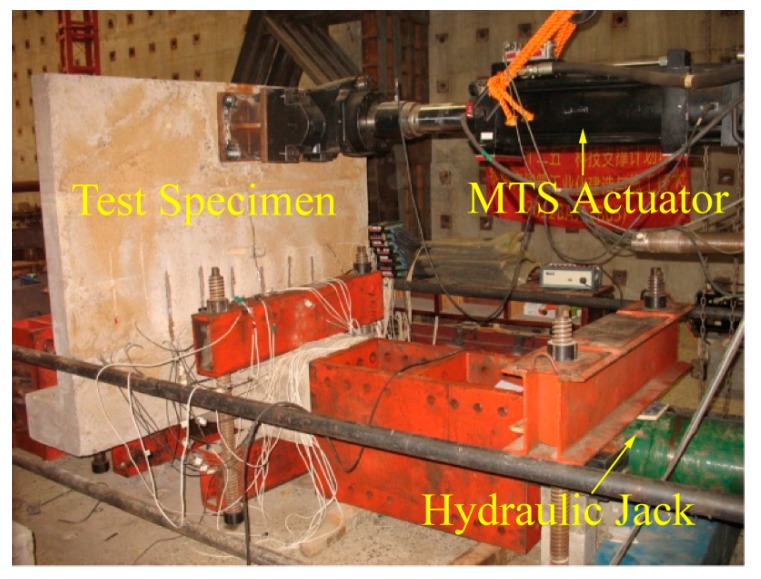
The test site.

**Figure 16:**
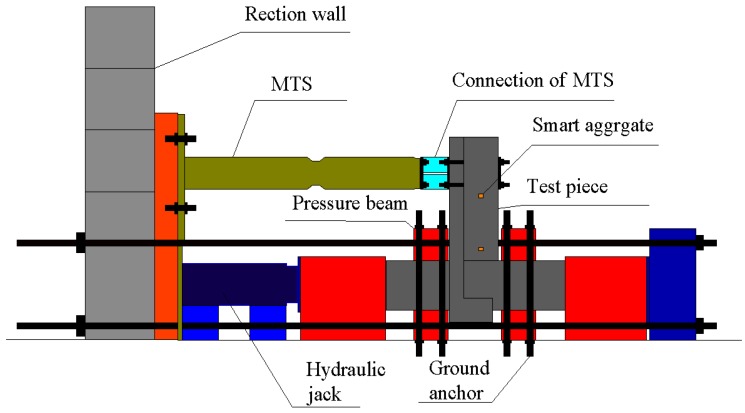
Loading device.

To verify the developed active CCD function of the system, a reinforced concrete (RC) beam-column joint with a partial RC floor embedded with WSAs is selected as the object of the validation test. The dimensions and the test site are shown in Figure 14 and Figure 15, respectively. WSAs are embedded in the specimen before the casting of the concrete. Two WSAs are separately arranged at 100 mm and 600 mm to the beam’s end, as shown in Figure 16.

**Figure 17:**
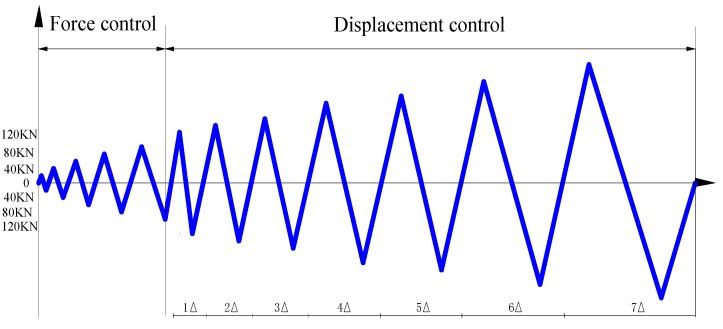
Loading scheme.

The main devices of the test include a reaction wall, an MTS 50 t loading system, and a 200 t hydraulic jack. During the test, the actuator and hydraulic jack are connected horizontally on the reaction wall, and then the specimen is installed horizontally. The column end of the specimen is connected with the hydraulic jack, and the other end is connected with the end plate. Through the pull rod, the reacting-force wall, the hydraulic jack, the specimen, and the end plate are connected. The MTS actuator is linked with the specimen through an adapting piece. The diagram of the loading device is shown in Figure 16. During the loading process, the vertical axial load of the column is applied through the 200 t hydraulic jack. Through the voltage stabilizer and strain gauge, the vertical load is maintained at about 80 t during the test. Meanwhile, the horizontal pseudo-static load is applied by the 50 t actuator on the end of the beam. The first step is applying 20 kN pre-load for the validity check of the component and other facilities. During the test, the combination loading plan of the force control and displacement control is adopted as shown in Figure 17. When the specimen is in the elastic stage, the force control loading plan is used, with the increment of force of 20 kN for one cycle in every loading level; when the specimen yields, the loading plan switches to displacement control. The displacement increment is 1Δ, namely Δ/Δ_y_ = ±1, ±2, ±3, ±4 (where Δ is the applying lateral displacement, and Δ_y_ is the yield displacement), for one cycle in every level, until the applying force falls to 80% of the bearing capacity or the specimen is directly destroyed.

The test takes advantage of a Structural Health Monitoring System based on WSAs to perform real-time monitoring. To increase the sensitivity to concrete cracks [9], a sweep sine wave is selected as the excitation signal. The amplitude is 10 V, the duration is 10 s, and the frequency progressed from 100 Hz to 10 kHz. When the specimen is loaded to a certain level, the loading state is maintained for several minutes. During this time, the signal generator (NI9074) produces the excitation signal on one of the smart aggregates, while at the same time the other smart aggregates function as sensors. Then, the specimen returns to the original position and the actuator stops loading. For each loading level, the measurements are continuously carried out 10 times before switching to the next loading level.

**Figure 18:**
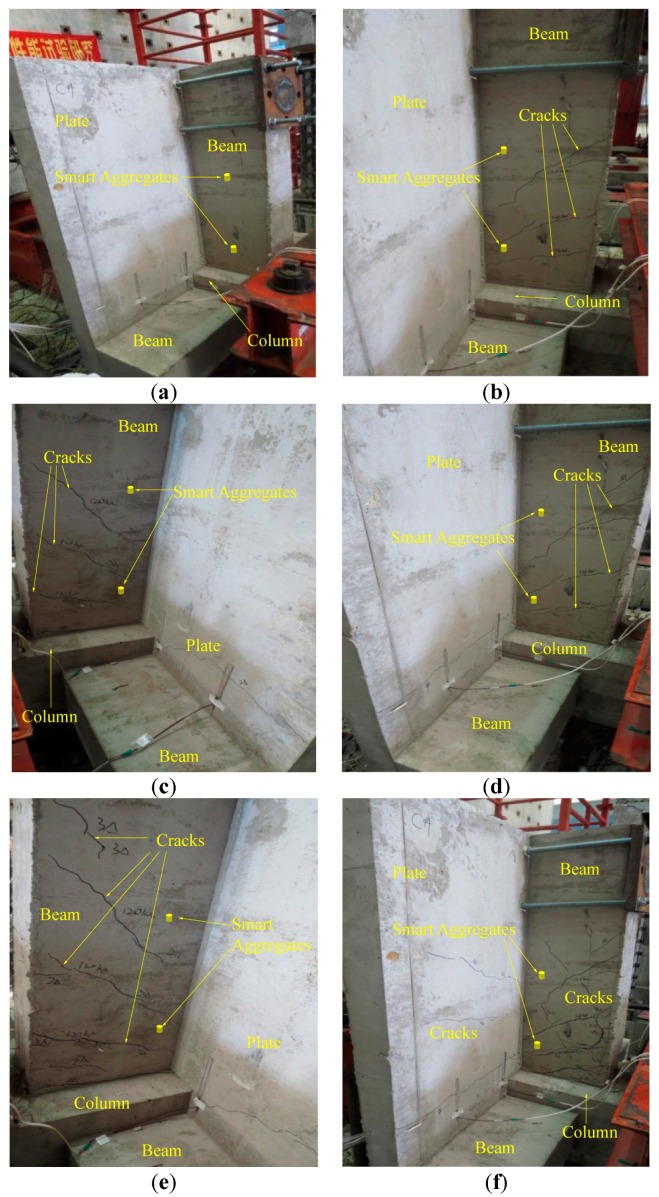
The different damage states of the specimen. (**a**) The original health status; (**b**) 1△; (**c**) 2△; (**d**) 3△; (**e**) 4△; (**f**) 5△.

**Figure 19:**
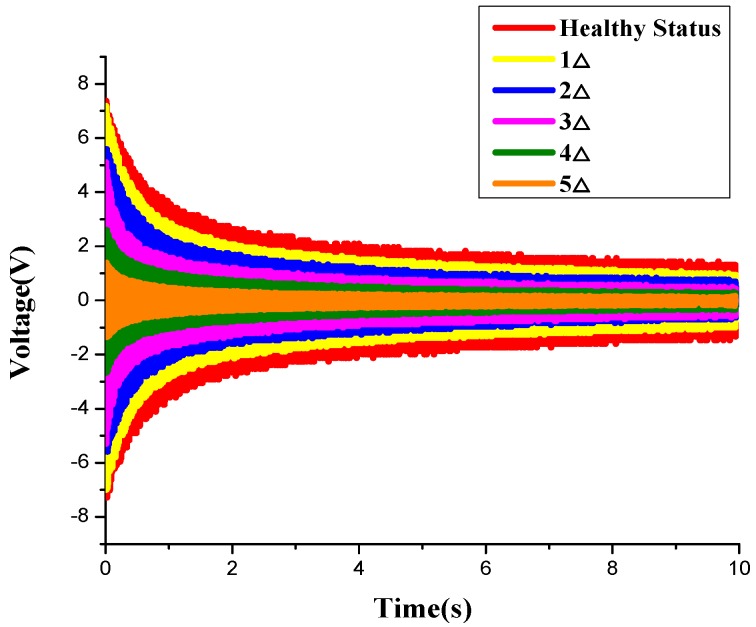
The variation of voltage value of the smart aggregates (SAs) under different damage states.

**Figure 20:**
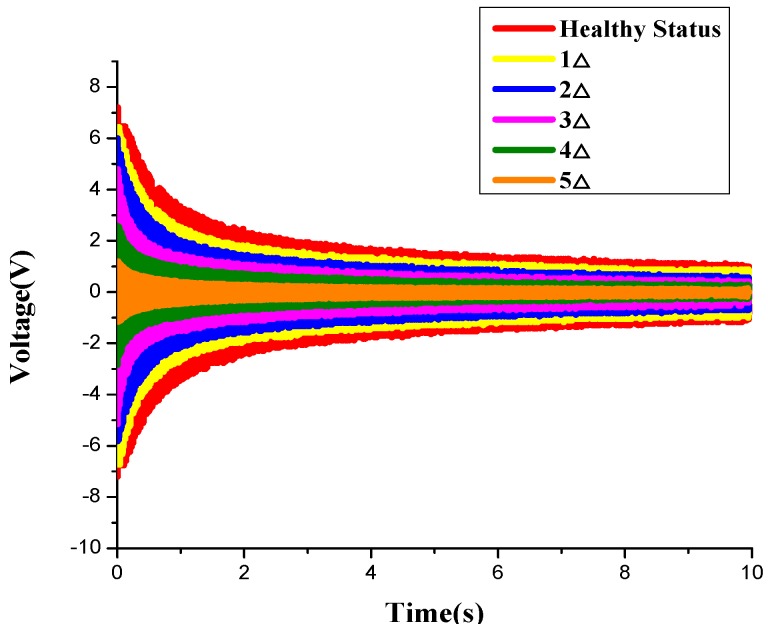
The variation of voltage value of the WSAs under different damage states.

**Figure 21:**
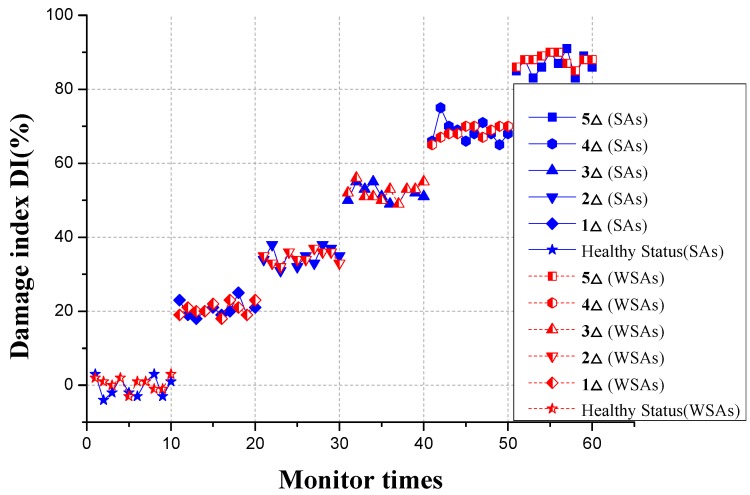
Damage index in different conditions.

In the experiment, both the WSA and the original wired smart aggregate are used. As shown in Figure 18, Figure 19 and Figure 20, respectively, with the development of the crack inside the specimen, the amplitudes of the monitoring signals decrease gradually. The damage indices at each loading level throughout the test process are shown in Figure 21. It can be seen that, as the structure progresses into different damage states, the corresponding damage index changes drastically to different levels. The increase of the damage index reflects that the structure has reached a new damage level. The experiment validates that the developed system can effectively monitor and identify the crack damage status inside the concrete structure. The result of the WSA is consistent with the wired smart aggregate [37].

## 4. Conclusions

In this paper, a wireless smart aggregate-based concrete crack detection (CCD) system was designed and developed. Experiments were performed to validate the efficiency of the proposed system. The experimental results demonstrate that the developed system can not only effectively identify the development of crack damage inside concrete structures, but also realize the online CCD of concrete internal stress change. Moreover, the developed system is “wire-free” and power efficient, which makes it suitable for the complex environment of a construction site. This work provides important support for practical large scale concrete structural health monitoring, and helps to promote smart aggregate technology for SHM from the laboratory research level to a practical engineering application level.

## Figures and Tables

**Table 1:** Comparison of several wireless network technologies.

Category	Zigbee	Bluetooth	Wi-Fi	Tele Communication
Single point coverage range	50–300 (m)	10 (m)	50 (m)	Several (km)
Network expansion capabilities	Automatic	None	None	Relying on existing network coverage
Complexity	Low	Medium	High	Medium
Time for network connection	30 s	10 s	3 s	Several seconds
Fee	None	None	None	High
power consumption	Low	High	High	Medium
Data rate	250 Kbps	1 Mbps	1 to 11 Mbps	Norma1ly 19.2 Kbps
